# Curcumin as an adjunct in periodontal therapy: local effects, delivery systems, and clinical perspectives

**DOI:** 10.3389/fdmed.2026.1861025

**Published:** 2026-09-03

**Authors:** Ana Carolina Aparecida Rivas, Mario Taba, Paola Gomes Souza

**Affiliations:** Department of Oral and Maxillofacial Surgery and Traumatology, and Periodontology, School of Dentistry of Ribeirão Preto – University of São Paulo, Ribeirão Preto - Sp, Brazil

**Keywords:** biofilm, curcumin, periodontal disease, plant-based therapy, turmeric

## Abstract

Curcumin, the active polyphenol in turmeric (Curcuma longa), is well-known for its anti-inflammatory, antioxidant, and antimicrobial properties, making it a promising therapeutic agent for a range of diseases, including neuroinflammatory conditions and periodontal disease. Periodontitis, a prevalent oral disease, is associated with chronic inflammation and bacterial infection. Curcumin has demonstrated antibacterial effects against periodontal pathogens such as Aggregatibacter actinomycetemcomitans, Porphyromonas gingivalis, and Tannerella forsythia when applied locally as a sub-gingival gel in conjunction with scaling and root planning (SRP). Furthermore, curcumin's ability to produce reactive oxygen species (ROS) under visible light exposure enhances its effects through photodynamic therapy (PDT) and potentiates its antibacterial and anti-inflammatory actions. Curcumin demonstrates promising potential as an adjunctive therapy for periodontitis, indicating that it may serve as a valuable adjuvant strategy for improving periodontal management. Nonetheless, additional well-designed clinical and translational studies are warranted to confirm these benefits.

## 1 Introduction

### 1.1 Overview of curcumin and potential applications

**Figure 1:**
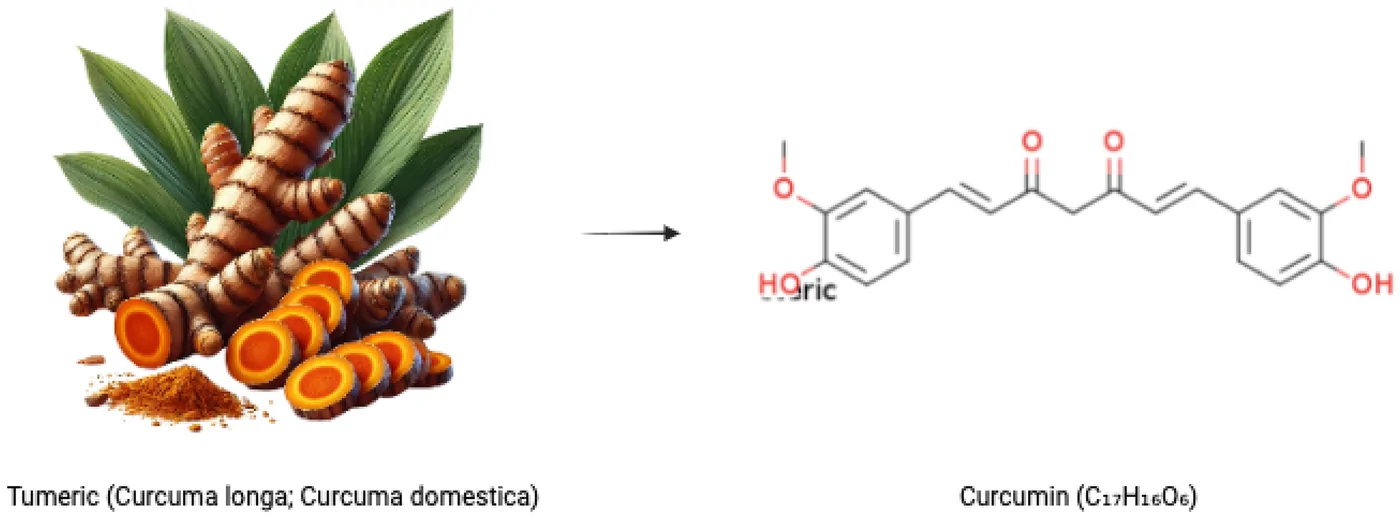
Turmeric, a spice from the Curcuma longa root, belongs to the ginger family. Its main compound, curcumin (C_17_H_16_O_6_), is a polyphenol with antioxidant and anti-inflammatory properties. Image of turmeric generated with DALL·E 3.

Plant-based therapies are used in various cultures around the world, and there is a belief that herbal medicines provide health benefits without causing adverse effects. Plant-derived medications are already widely used, and some nations allocate 40% to 50% of their healthcare budget to developing new drugs. Turmeric (*Curcuma longa*), also known as curcuma, is a plant native to South Asia, particularly India, and tropical countries like Indonesia and China. For centuries, it has been used in Ayurvedic medicine and cuisine, being an essential ingredient in curry. Its main active compound, Curcumin (diferuloylmethane), is recognized for its anti-inflammatory and antioxidant properties, sparking scientific and industrial interest in its potential therapeutic applications. Despite the difference in names—curcuma in Portuguese-speaking countries and turmeric in English-speaking regions—both refer to the same root, widely used in various cultures (Figure 1).

Curcumin exhibits potent antitoxic effects by neutralizing harmful substances and enhancing the liver's detoxification processes. Its anti-inflammatory properties are well-documented, as it modulates key inflammatory pathways, offering benefits in conditions like arthritis and cardiovascular diseases. As a powerful antioxidant, curcumin scavenges free radicals and protects cells from oxidative stress, which plays a role in aging and degenerative diseases like Alzheimer's. Furthermore, curcumin demonstrates anticarcinogenic and antimutagenic activities, preventing cancer development by regulating various molecular pathways involved in cell cycle, apoptosis, and angiogenesis. While it has shown antifertility effects in animal studies, its antibacterial, antifungal, and antiviral properties make it effective against a range of pathogens, including *Staphylococcus aureus* and *Hepatitis C*. Additionally, curcumin aids in the prevention and healing of gastric ulcers by enhancing mucosal defense and balancing gastric acids. Its hypocholesteremic effects help lower harmful cholesterol levels and promote cardiovascular health. Given these diverse biological effects, curcumin holds significant promise as a therapeutic agent (1–9), particularly in the context of periodontal disease (PD), where its anti-inflammatory, antimicrobial, and antioxidant properties can play a crucial role in managing gum inflammation, preventing infection, and promoting tissue healing.

In the treatment of PD, its anti-inflammatory action consists of modulating the extracellular matrix (ECM), inhibiting inflammatory mediators such as NO, PGE (2), IL-6, IL-8, and MMP-3, which are associated with ECM degradation, and reducing the release of *(35)S-GAG* (10). Additionally, Cur promotes ECM biosynthesis and TGF-ß1, suggesting its potential to control inflammation and improve tissue regeneration (11). Thus, this review aims to provide a comprehensive understanding of the use of curcumin in the treatment of periodontitis, exploring its mechanisms of action, therapeutic potential, and limitations compared to conventional treatments widely used in clinical practice, as well as discussing its prospects as an adjuvant in periodontal disease management.

### 1.2 Potential systemic implications (experimental) of curcumin

The systemic implications of curcumin are based on its vast range of properties, including: antioxidant, anti-inflammatory, neuroprotective, anticancer, hepatoprotective, cardioprotective, immunomodulatory, antifertility, antimicrobial, antiallergic, antidermatophytic, and antidepressant effects (12). In the West, it is used to relieve hives, hepatitis, dermatitis, sore throat, and joint inflammation, as well as being considered an aromatic stimulant and relieving bloating and abdominal discomfort (13). Among this vast potential for use, we have the treatment of systemic conditions such as: gastrointestinal disorders, respiratory disorders, inflammatory processes, diabetes mellitus, cardiovascular diseases, hepatotoxic diseases, brain injuries, oxidative stress in general, allergies, and neoplasms (14, 15).

However, limitations still exist regarding the use and experimentation of curcumin application, such as its low water solubility and low chemical stability, unstable oral bioavailability, and unpredictable cellular absorption. This necessitates further studies and the development of strategies beyond controlled-rate administration, slow release, and targeted application, such as the production of nanoformulations. Therefore, an in-depth study of C. longa is necessary, focusing on its taxonomic classification, traditional uses, botanical description, phytochemical components, pharmacology, toxicity, and safety aspects related to its main compound, curcumin, to explore trends and perspectives for future research (16, 17).

Despite promising evidence regarding the systemic applications of curcumin, limitations remain regarding evidence from clinical trials proving its efficacy as an adjuvant therapy. In this context, further preclinical and clinical studies are necessary to deepen the investigation of this compound. Furthermore, it is essential to ensure the rigorous analysis, standardization, and validation of its therapeutic potential, as well as its safety and efficacy, before curcumin-based medications can be incorporated into the market as first-line treatment options (18, 19).

### 1.3 Periodontal disease: pathogenesis, prevalence, and treatment strategies

PD is a long-term inflammatory condition affecting the periodontium, with its severe stage marked by the loss of the periodontal ligament and the deterioration of the surrounding alveolar bone (20). It typically begins with gingival inflammation (gingivitis) caused by bacterial biofilms (dental plaque) and, if left untreated, can progress to periodontitis, a more severe form of the disease. In advanced stages, periodontitis leads to progressive destruction of the periodontal ligament and alveolar bone, resulting in tooth mobility and eventual tooth loss. The inflammatory response plays a key role in this destruction, as immune cells release cytokines and matrix metalloproteinases (MMPs), which contribute to tissue breakdown. Risk factors such as poor oral hygiene, smoking, diabetes, genetic predisposition, and aging further exacerbate the disease. Given its association with systemic conditions like cardiovascular disease and diabetes, periodontal disease is not only an oral health issue but also a significant public health concern (21).

The worldwide occurrence of periodontal disease is projected to rise in the coming years due to the growing aging population and the higher retention of natural teeth, resulting from a notable decline in tooth loss among older individuals. Globally, PD impacts approximately 20% to 50% of the population (22). The primary objective in treating periodontitis is to achieve and sustain effective infection control in the dentogingival region. This is accomplished through scaling and root planning, which, when combined with proper self-care for supragingival plaque control, helps modify the subgingival environment by disrupting the microbial biofilm and reducing inflammation (23). Patients with systemic diseases and periodontitis can benefit from non-surgical periodontal therapy (NSPT), which has been shown to reduce probing depth and bleeding, with results comparable to those of healthy patients (24).

#### 1.3.1 Periodontal treatment, possible results and limitations

The treatment of PD is primarily done through mechanical debridement. This approach includes using polishing brushes, air-abrasive instruments, manual and ultrasonic scalers, as well as performing scaling and root planning (SRP) (25). Although significant efforts are made to remove microorganisms, the complex structure of tooth surfaces makes total eradication difficult (26). Therefore, additional strategies, including antibiotics, antiseptics, laser therapy, and antimicrobial photodynamic therapy have been integrated with mechanical debridement to enhance treatment effectiveness in periodontics.

Chemical agents have been employed not only to extend and enhance the effects of the mechanical treatments in periodontitis but also, in some cases, to facilitate or even serve as an alternative to such procedures, for example, the scaling and root planning associated with local application of Chlorhexidine (CHX). Many studies suggest that CHX is regarded as the most potent antimicrobial mouthwash (27, 28). CHX binds to bacterial membranes, increasing permeability and preventing adhesion. Subgingival irrigation with CHX (0.02%–2.0%) reduces probing depth (PD) (29), with 2.0% being the most effective, sustaining results for up to 24 weeks. However, side effects such as staining, taste alteration, and mucosal erosion may limit its use (30).

#### 1.3.2 Antibiotics and periodontal diseases

Another strategy of treatment is root planning with tetracycline. Tetracyclines, particularly doxycycline, are widely used in the treatment of periodontitis due to their high lipid solubility and long half-life (31, 32), which enhances their penetration into biological fluids, including gingival crevicular fluid (33, 34). Systemic doxycycline not only reduces inflammation in periodontal tissues but also inhibits the activity of metalloproteinases, leading to a reduction in periodontal parameters like probing depth (35). Furthermore, it possesses antimicrobial properties that contribute to its effectiveness in managing periodontal disease (36).

Although systemic therapy is effective, it has drawbacks such as gastrointestinal side effects, photosensitivity, and the risk of bacterial resistance. In addition to the present side effects, a study confirms that systemic doxycycline, when added to scaling and root planning (SRP) in diabetic patients with periodontitis, does not improve metabolic control (HbA1c) or periodontal outcomes (clinical attachment levels, CAL) after 3 months compared to SRP alone (35). Another strategy is SRP associated with Metronidazole MTZ, which is a widely used antibacterial medication in clinical settings, known for its ability to disrupt bacterial protein synthesis (37, 38). However, this can contribute to the development of bacterial resistance and is also associated with common gastrointestinal side effects, such as nausea, vomiting, heartburn, and reduced appetite during treatment (39).

MTZ is particularly effective against anaerobic pathogens like *Porphyromonas gingivalis*, which is often eliminated from periodontal pockets through its action (40). It has been shown to result in a significant improvement on clinical outcomes in periodontitis treatment, including increased attachment gain and reduced probing depth at deeper sites when compared to mechanical debridement alone (40, 41). A study has found that MTZ does not directly enhance the killing of *P. gingivalis* by PMNs but rather modulates their activity (39). Specifically, MTZ reduces phagocytosis and ROS production induced by *P. gingivalis* (39) in its lowest concentrations (2 μg/ml) within the regular therapeutic dosages. These findings suggest that MTZ's role may lie in altering PMN functions, creating a more hostile environment for the bacteria through mechanisms such as degranulation and NETosis. MTZ is commonly prescribed alone or in combination with amoxicillin for managing periodontitis, enhancing its bactericidal efficacy, and could be used in gel or tabs to treat periodontitis in chronic conditions (42, 43).

Another recent study showed that MTZ, in both gel and tablet forms, combined with scaling and root planning (SRP), improves clinical parameters such as CAL, PPD, and BOP. However, in advanced periodontitis cases (beyond Stage II and Grade B), it does not provide additional benefits over SRP alone, which remains the *criterium aureum* for periodontal treatment (44). The ideal antibiotic regimen should carefully balance therapeutic efficacy with the risk of adverse effects, which may compromise treatment success by reducing patient adherence and promoting antibiotic resistance (45). Factors such as dosage, duration, and patient-specific characteristics must be considered to optimize outcomes while minimizing side effects. Additionally, the indiscriminate use of antibiotics can disrupt the host microbiota, potentially leading to secondary infections or microbial dysbiosis, further underscoring the need for a judicious approach in clinical decision-making.

#### 1.3.3 Antimicrobial photodynamic therapy (aPDT) and laser therapy for periodontal disease treatment

Since the 1980s, photo therapy lasers have been utilized in periodontics, with initial reports highlighting their role in periodontal surgery (46). These lasers are generally categorized into two types: high-power and low-power lasers. High-power lasers, such as Er:YAG, CO₂, Nd:YAG, and Er,Cr:YSGG, are used for the removal of inflamed gingival tissue, decontamination of periodontal pockets, tissue vaporization, and bone cutting. Low-power lasers, such as the diode laser and low-level laser therapy (LLLT), are employed to stimulate tissue regeneration, reduce pain and inflammation, accelerate healing, and assist in antimicrobial photodynamic therapy.

Antimicrobial Photodynamic Therapy (aPDT) and laser therapy are techniques used in periodontal treatment. The aPDT involves the use of a photosensitizing agent—such as methylene blue or toluidine blue—combined with a light source, typically a laser or light-emitting diode (LED), to target and destroy periodontal pathogens. The photosensitizer binds to bacterial cells and, when activated by the light, produces reactive oxygen species that kill the bacteria. This process also helps in detoxifying endotoxins and reducing inflammation in the treated area (47, 48). Laser therapy, on the other hand, generally refers to the use of high-power lasers (like Er:YAG, CO₂, or Nd:YAG) or low-power lasers (like diode lasers) to directly target tissues for various purposes, such as cutting, coagulating, or stimulating healing. In periodontal therapy, lasers are used for tissue debridement, bacterial elimination, and enhancing tissue regeneration (49, 50). In periodontal disease treatment, both approaches are often used together. For example, aPDT can be combined with mechanical scaling and root planning (SRP) or other laser therapies to improve outcomes by effectively reducing microbial load and enhancing tissue healing (46–51).

The mechanism behind aPDT involves a photosensitizing agent that selectively attaches to the target cell and, upon exposure to light of an appropriate wavelength, undergoes activation. This process generates reactive oxygen species, leading to microbial destruction while minimizing damage to surrounding tissues. When combined, aPDT and laser therapy can exhibit a synergistic effect, as laser treatment promotes tissue regeneration and decontamination, while aPDT utilizes a light-activated photosensitizer to eliminate periodontal pathogens. Together, these approaches enhance healing by modulating the inflammatory response, leading to improved periodontal outcomes (52). Studies have demonstrated that incorporating sPDT as an adjunct to SRP, whether in non-surgical or surgical phases of periodontal treatment, leads to significant improvements in clinical parameters (BOP, CAL, and PD) (53–56). However, a 2012 study by Novaes et al. demonstrated that SRP was more effective than aPDT in reducing the prevalence of Red Complex periodontal pathogens (57). Furthermore, the study noted bacterial recolonization in aPDT-treated areas, particularly involving T. forsythia and P. gingivalis.

### 1.4 Curcumin as an adjunct in periodontal therapy: anti-inflammatory and regenerative potential

In this context, curcumin, the active compound in turmeric, has gained attention as an adjunct therapy due to its anti-inflammatory, antimicrobial, and antioxidant properties. Studies suggest that curcumin can enhance periodontal treatment outcomes by further suppressing inflammation, inhibiting bacterial growth, and promoting tissue healing, making it a promising natural alternative in periodontal therapy (57, 58). In addition, a study has demonstrated that curcumin inhibits the production of IL-6 and IL-8, two cytokines present in a periodontal inflammation (10). IL-6 levels are significantly higher in patients with periodontitis compared to healthy controls, and these levels decrease after periodontal treatment. IL-6 works synergistically with other cytokines like TNF-α and IL-1β to promote the inflammatory cascade in periodontitis. Other cytokines like IL-8 are involved in attracting neutrophils to the inflamed site, intensifying the inflammatory process (59).

**Figure 2:**
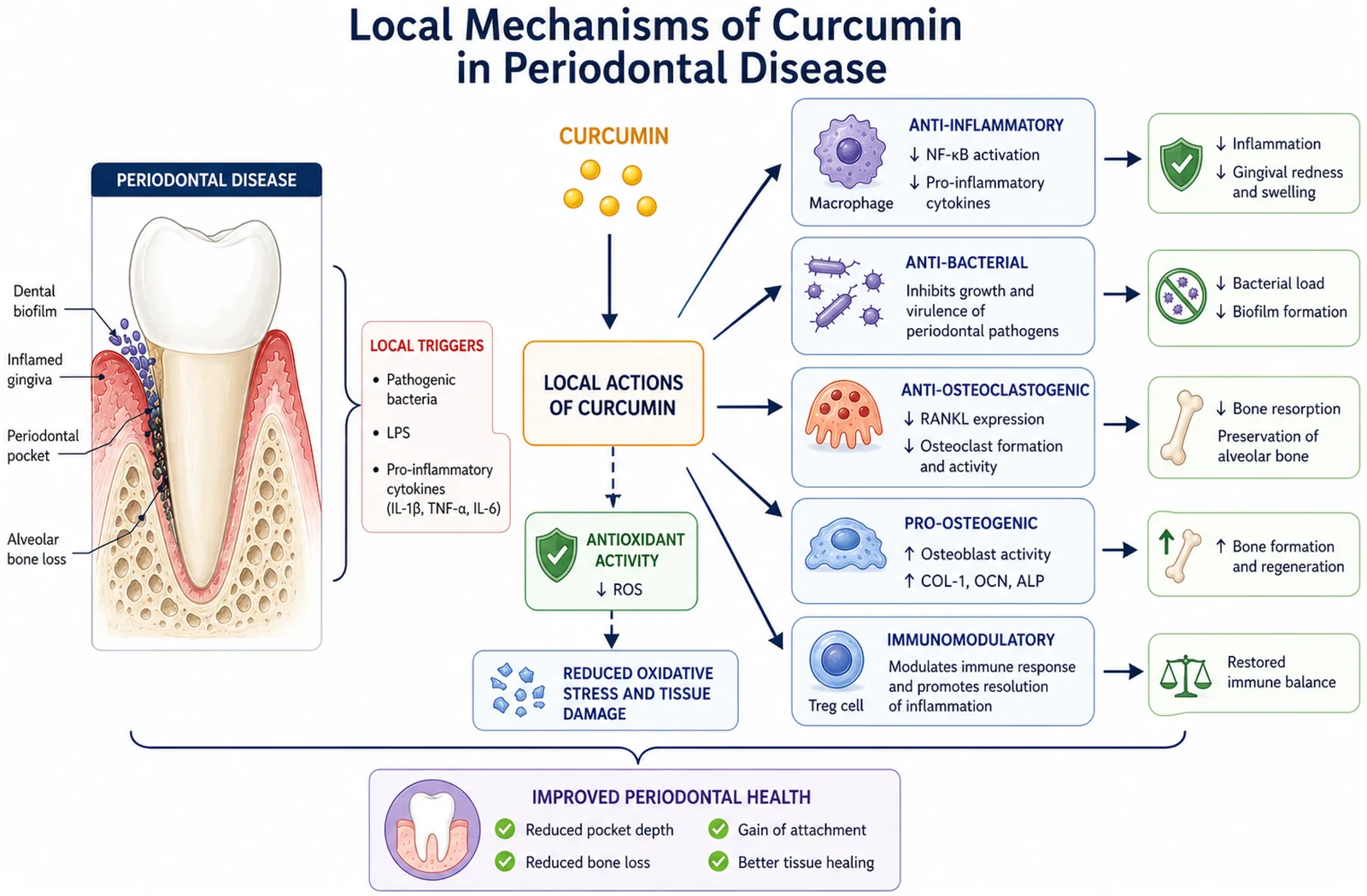
Local mechanisms of curcumin in periodontal disease. Curcumin exerts multiple local therapeutic effects in periodontal tissues by modulating inflammatory, microbial, oxidative, and bone remodeling pathways. Its anti-inflammatory action includes inhibition of NF-*κ*B activation and reduction of pro-inflammatory cytokines such as IL-1β, TNF-α, and IL-6, leading to decreased gingival inflammation. Curcumin also demonstrates antibacterial activity by reducing periodontal pathogen growth and biofilm formation. In addition, it suppresses osteoclastogenesis through downregulation of RANKL expression and osteoclast activity, while promoting osteogenesis by enhancing osteoblast function and increasing the expression of bone-related markers such as COL-1, OCN, and ALP. Its antioxidant and immunomodulatory properties contribute to reduced oxidative stress and restoration of immune balance, ultimately resulting in improved periodontal health, reduced pocket depth, preservation of alveolar bone, and better tissue healing. Image generated with DALL·E 3.

Curcumin, by inhibiting the activation of NF-*κ*B, a transcription factor activated by IL-1β, and reducing the expression of MMP-3, an enzyme that degrades the extracellular matrix, restores collagen synthesis and protects human chondrocytes (60). This mechanism plays a crucial role not only in osteoarthritis but also in periodontitis, as its chronic pathogenicity leads to the destruction of periodontal tissues. Such modulation of pro-inflammatory cytokines may reduce extracellular matrix degradation and promote tissue regeneration, making curcumin a potential complementary therapeutic agent in the treatment of periodontitis (Figure 2).

Previous research has demonstrated the antibacterial properties of curcumin against several oral pathogens, including *Streptococcus mutans*, *Lactobacillus casei*, *Actinomyces viscosus*, *Porphyromonas gingivalis*, *Prevotella intermedia*, *Fusobacterium nucleatum*, and *Treponema denticola* (61, 62), and recent research has demonstrated significant antibacterial and anti-biofilm properties of curcumin against periodontal pathogens, particularly *Porphyromonas gingivalis*, a key bacterium involved in periodontitis. Studies indicate that curcumin can inhibit bacterial adhesion and biofilm formation in a dose-dependent manner and it reduces the expression of genes coding for major virulence factors such as fimA, hagA, and rgpA, effectively attenuating the virulence of *P. gingivalis* (63). These findings suggest that curcumin's multifaceted actions, including its antibacterial effects and ability to disrupt biofilm formation, could serve as a promising cost-effective therapeutic strategy for periodontal disease.

Several studies suggest that curcumin could serve as an effective adjuvant therapy. For instance, research evaluating the effects of photodynamic therapy with curcumin against *P. gingivalis* and *Aggregatibacter actinomycetemcomitans* demonstrated that the combination of curcumin and blue LED significantly reduced *A. actinomycetemcomitans*, while *P. gingivalis* showed lower susceptibility. Additionally, chlorhexidine 0.12% exhibited a weaker bactericidal effect. These findings indicate that PDT with curcumin holds promise, particularly against *A. actinomycetemcomitans*, though further studies are needed (64). Another study developed a curcumin-based microemulsion mouthwash for oral disinfection in combination with photodynamic therapy. In *in vitro* tests, the combination of curcumin and PDT significantly reduced biofilms of *Candida albicans*, methicillin-resistant *Staphylococcus aureus* (MRSA), and *Escherichia coli*. The results indicate that the formulation is promising but requires further evaluation in clinical trials (65).

Other results showed that curcumin, both as an irrigant and in aPDT with blue LED, improved short-term periodontal clinical parameters (probing depth reduction and clinical attachment level gain) compared to SRP alone (66). Research investigating the treatment of experimental periodontitis in rats has demonstrated that curcumin, either on its own or combined with photodynamic therapy (aPDT), can influence inflammation (67) and reduce the loss of alveolar bone (67–69). Some studies have utilized modified curcumin and demonstrated that it outperforms natural curcumin in terms of bone absorption. Studies show that while natural curcumin (30–100 mg/kg/day) (69–71) had mixed effects on alveolar bone loss, chemically modified curcumin (30 mg/kg/day) consistently reduced it (69–75). This is because modified curcumin is a more potent inhibitor of MMPs, and only the modified form has been shown to significantly reduce alveolar bone loss (72–76).

However, despite these promising findings, there is growing recognition of important limitations that may restrict the clinical applicability of curcumin. A key concern is its low bioavailability, as consistently demonstrated in both animal and human pharmacokinetic studies (68, 80). Following oral administration, curcumin exhibits poor gastrointestinal absorption, resulting in extremely low or even undetectable plasma concentrations, even at high doses (77, 78). Furthermore, increasing the administered dose does not necessarily lead to proportional increases in systemic levels, suggesting inherent limitations in absorption (79). In addition, curcumin appears to be largely retained within the gastrointestinal tract, with only trace amounts reaching peripheral tissues such as the liver and kidneys (77, 80). The route of administration also plays a crucial role, as parenteral delivery methods yield higher plasma concentrations than oral intake, although these levels decline rapidly over time (78, 79). Another important factor is its extensive metabolism: once absorbed, curcumin is rapidly converted into conjugated forms, such as glucuronides and sulfates, as well as reduced metabolites, which further limit the availability of the active compound (80, 81).

Beyond pharmacokinetic challenges, the heterogeneity of formulations, dosages, and delivery systems across studies complicates the comparison of results and the establishment of standardized therapeutic protocols (82). Moreover, most of the available evidence is derived from preclinical models, with a limited number of well-controlled clinical trials confirming its efficacy in humans (83). Although chemically modified curcumin has shown greater potency, particularly due to enhanced MMP inhibition, its long-term safety and potential off-target effects remain insufficiently investigated (74, 76).

Therefore, additional research and well-designed clinical trials are essential to better elucidate the effects of curcumin on immune cell activity and cytokine expression within gingival tissues, particularly in the context of host immune response modulation. A deeper understanding of these mechanisms may support the development of more targeted and effective therapeutic strategies aimed at modulating inflammation while minimizing systemic side effects. Ultimately, such approaches could improve periodontal treatment outcomes by promoting tissue repair and reducing alveolar bone loss. Curcumin has been investigated through multiple delivery systems in periodontal therapy, ranging from topical formulations to advanced nanotechnology-based approaches. However, despite promising results, most of these strategies remain experimental, with limited translation into standardized clinical practice.

The data summarized above in the table highlight the wide range of curcumin delivery systems that have been investigated in periodontal therapy, spanning from conventional topical formulations to advanced nanotechnology-based approaches. While several of these strategies, particularly local delivery systems such as gels, chips, and aPDT, have demonstrated promising effects on clinical and inflammatory parameters, their translation into routine clinical practice remains limited. Notably, most commercially available formulations are restricted to systemic supplements or non-standardized oral care products, which do not specifically target periodontal tissues and are often associated with suboptimal bioavailability (Table 1).

**Table 1:** Current curcumin delivery strategies highlight a significant gap between experimental efficacy and clinical applicability.

Study Author/ Year	Delivery system/formulation	Experimental/Clinical application	Sample size	Commercial availability	Limitations/Barriers to clinical Use
Behal et al. & Anitha et al. (84, 85)	Topical gel (intrapocket)	Applied after SRP; improves probing depth (PD) and clinical attachment level (CAL)	*n* = 30/*n* = 20	Limited/not standardized	Lack of standardized concentration, dosage frequency, and formulation stability
Waghmare et al. & Pulikkotil et al. (86, 87)	Mouthwash	Reduces plaque accumulation and gingival inflammation; comparable to chlorhexidine in some studies	*n* = 100/–	Partially available (natural products)	Low substantivity and limited bioavailability
Kandwal et al. (88)	Periodontal irrigant	Used as an adjunct during SRP procedures	*n* = 60	Not standardized	Insufficient clinical evidence and lack of defined protocols
Jain et al. (89)	Local delivery systems (chip/strip)	Inserted into periodontal pockets for sustained release (similar to PerioChip)	–	Experimental	Need for long-term clinical trials and regulatory approval
Pourabbas et al. & Birang et al. (90, 91)	Antimicrobial photodynamic therapy (aPDT)	Curcumin used as a photosensitizer with blue LED	*n* = 22/*n* = 20	Experimental	Inconsistent results and lack of protocol standardization
Zambrano et al. (92)	Nanocurcumin (local delivery)	Nanoparticles enhance anti-inflammatory and bone-protective effects	*n* = 16	Not clinically available	Safety concerns, cost, and lack of human studies
Chainani-Wu. (93)	Nanocurcumin (systemic)	Oral administration targeting systemic inflammation	–	Available as supplements	Variable absorption and limited periodontal-specific evidence
Liu et al. (94)	Biomaterials/membranes (GBR)	Curcumin incorporated into scaffolds for tissue regeneration	–	Experimental	Lack of clinical validation in humans
Bansal et al. (95)	Local sponges/films	Controlled release systems applied to gingival tissues	–	Not commercialized	Limited standardization and clinical validation
Anand et al. & Hewlings et al. (77, 82)	Oral capsules (systemic use)	Dietary supplementation with systemic anti-inflammatory effects	–/–	Available	Poor bioavailability and indirect periodontal effects
Curylofo-Zotti et al. & Wang et al. (72, 76)	Chemically modified curcumin (CMC)	Enhanced inhibition of MMPs and improved biological activity	*n* = 60/*n* = 20	Experimental	Safety profile, regulatory approval, and lack of clinical trials

In contrast, more sophisticated approaches, including nanocurcumin and chemically modified curcumin, have shown enhanced biological activity and improved therapeutic potential in preclinical models, especially in terms of inflammation control and inhibition of matrix metalloproteinases. However, these systems are still largely experimental and face significant barriers, including limited clinical validation, safety concerns, and regulatory challenges. Additionally, the marked heterogeneity in study design, drug concentrations, delivery protocols, and outcome measures further complicates the comparison of results and the establishment of standardized therapeutic guidelines.

Collectively, these findings reveal a clear translational gap between experimental efficacy and clinical applicability. Despite the growing body of evidence supporting the biological effects of curcumin, the lack of well-designed randomized clinical trials and standardized delivery systems continues to hinder its incorporation into evidence-based periodontal therapy. Future research should therefore focus on optimizing delivery strategies that enhance bioavailability and tissue targeting, while also ensuring safety and reproducibility, ultimately facilitating the integration of curcumin into clinically relevant treatment protocols.

### 1.5 Future perspectives: strategies to enhance curcumin stability and therapeutic efficacy

Despite these promising results, further studies are required to optimize curcumin's bioavailability and ensure its long-term efficacy in periodontal therapy. One of the major challenges limiting its clinical application is its intrinsic chemical instability and rapid degradation under physiological conditions. Curcumin is highly sensitive to pH, light, and enzymatic metabolism, which significantly reduces its bioactive concentration at the target site. To address these limitations, future research should focus on advanced delivery systems, including nanoparticles, liposomes, micelles, and polymer-based encapsulation strategies, which have shown potential to enhance stability, absorption, and tissue penetration (96–99). Notably, chemically modified forms of curcumin appear to exhibit superior efficacy, particularly in preserving bone structure through enhanced inhibition of matrix metalloproteinases. Nevertheless, despite these encouraging findings, significant challenges remain. Limitations related to poor bioavailability, rapid metabolism, and lack of standardized delivery systems continue to restrict its clinical applicability.

Advances in formulation strategies, including nanotechnology-based delivery systems and targeted therapeutic approaches, may help overcome these barriers by improving stability, absorption, and tissue-specific activity. Ultimately, if these pharmacokinetic and translational challenges are successfully addressed, curcumin has the potential to transition from an experimental adjunct to a clinically relevant tool in periodontal therapy. Its integration into modern periodontal care will depend on the development of safe, effective, and standardized protocols capable of delivering consistent and reproducible therapeutic benefits. In addition, combining curcumin with other therapeutic approaches represents a promising strategy to improve its biological effectiveness.

Synergistic effects have been observed when curcumin is used alongside conventional periodontal therapies, such as scaling and root planning (SRP), antimicrobial photodynamic therapy (aPDT), or biomaterial-based scaffolds (93, 100, 101). These combinations may enhance anti-inflammatory and antimicrobial outcomes while also improving bone preservation. Furthermore, a deeper understanding of the molecular mechanisms underlying curcumin's effects, particularly its role in modulating cytokine expression, oxidative stress, and matrix metalloproteinase activity, may help refine its clinical applications and minimize potential side effects through more targeted therapeutic strategies (102–104). Moreover, well-designed clinical trials with larger sample sizes and longer follow-up periods are essential to validate curcumin's efficacy and safety in real-world periodontal treatment. Ensuring biological effectiveness will depend not only on improving pharmacokinetic properties but also on developing standardized delivery protocols capable of achieving consistent therapeutic outcomes. Collectively, these advances may facilitate the transition of curcumin from a promising experimental compound to a clinically relevant adjunct in periodontal therapy.

## 2 Conclusion

Curcumin has shown strong potential as a complementary approach in periodontal therapy due to its anti-inflammatory, antioxidant, and antimicrobial effects, with studies demonstrating its ability to modulate inflammatory pathways, reduce periodontal pathogens, support tissue repair, and prevent alveolar bone loss, especially when used alongside conventional treatments such as scaling and root planning and antimicrobial photodynamic therapy. Chemically modified forms may offer enhanced efficacy, particularly in inhibiting matrix metalloproteinases and preserving bone structure. However, its clinical application remains limited by poor bioavailability, rapid metabolism, and the absence of standardized delivery systems, while the lack of long-term randomized clinical trials highlights the need for further research. Advances in nanotechnology-based formulations and targeted delivery strategies may overcome these challenges, enabling curcumin to become a safe, standardized, and clinically relevant tool in modern periodontal care.
